# The development of a multiplex recombinase polymerase amplification reaction to detect the most common causative agents of eumycetoma

**DOI:** 10.1007/s10096-025-05134-4

**Published:** 2025-04-30

**Authors:** Mickey Konings, Emmanuel Siddig, Kimberly Eadie, Carole Pab Minlekib, Maguette Faye, Doudou Sow, Ahmed Fahal, Annelies Verbon, Wendy van de Sande

**Affiliations:** 1 Erasmus MC, Department of Medical Microbiology and Infectious Diseases, University Medical Center Rotterdam, Dr. Molewaterplein 40, Rotterdam, 3015GD The Netherlands; 2 Faculty of Medical Laboratory Sciences, University of Khartoum, Kphartoum, Sudan; 3 Service de Parasitologie-Mycologie, UFR Sciences de la Santé, Université Gaston Berger, Saint-Louis, Senegal; 4 Mycetoma Research Center, University of Khartoum, Khartoum, Sudan; 5 Department of Internal medicine, UMC Utrecht, Utrecht, The Netherlands

**Keywords:** Mycetoma, *Madurella mycetomatis*, *Falciformispora senegalensis*, pan-fungal, RPA, point-of-care test

## Abstract

**Purpose:**

Mycetoma is a neglected tropical disease that affects the subcutaneous tissue. The disease can be caused by over 90 different pathogens, including both bacteria (actinomycetoma) and fungi (eumycetoma). While diagnostic tools for eumycetoma causative agents are available, these are generally not well suited for use in endemic regions. This study aims to develop an isothermal based multiplex recombinase polymerase amplification reaction (RPA), that can be integrated in the diagnostic workflow of endemic regions.

**Methods:**

The RPA was designed targeting the Internal Transcribed Spacer (ITS) region to detect the presence of fungal DNA, and to differentiate between *Madurella mycetomatis* and *Falciformispora senegalensis*. The performance of the RPA was evaluated using 71 fungal isolates and five actinomycetes reference isolates. Furthermore, the limit of detection (LOD) was determined for the different probes in singleplex and multiplex.

**Results:**

The ITS probe was positive for all 71 fungal isolates with a mean detection time of 13.1 min. The *M. mycetomatis* and *F. senegalensis* probes were only positive for their respective targets, with a mean detection time of 9.3 and 7.6 min, respectively. No cross-reactivity was detected, and a limit of detection of 0.01 ng of fungal DNA was found. The costs of the RPA ranged from €1.56 to €10.03, depending on the workflow.

**Conclusion:**

We developed a field-friendly multiplex RPA, that successfully detects fungal DNA and discriminates between *M. mycetomatis* and *F. senegalensis*. This tool holds promise for enhancing diagnostic capabilities in eumycetoma endemic regions, paving the way for improved patient management and treatment outcomes.

**Supplementary Information:**

The online version contains supplementary material available at 10.1007/s10096-025-05134-4.

## Introduction

Mycetoma is a neglected tropical disease (NTD), that presents as a chronic granulomatous infection affecting the subcutaneous tissue [1]. Clinically, it manifests as tumorous masses with formed sinuses that secrete purulent discharge containing biofilm-like structures known as grains [2]. Late-stage mycetoma, if left untreated, can lead to deformities, disabilities and in some cases, death [3]. Mycetoma is mainly prevalent in rural areas of tropical and subtropical regions, but is reported worldwide [4, 5]. The infection can be caused by both fungi and bacteria, referred to as eumycetoma and actinomycetoma, respectively. The two most frequent reported causative agents of eumycetoma are *Madurella mycetomatis* and *Falciformispora senegalensis*, accounting for 85% and 4% of reported eumycetoma cases, respectively [6].

In 2022, the World Health Organization stated that optimal management of eumycetoma includes rapid, point-of-care identification of the causative agents [7]. Classical diagnostic methods based on histology and culture of the infectious agents are time-consuming and not very accurate. The reported average times to identification were 204.4 h and 508.5 h, with a sensitivity of 93.8% and 84.5%, and a specificity of 42.8% and 85.7%, respectively [8]. In contrast, PCR based identification of *M. mycetomatis* has an average duration of 2.76 h with a 98.8% sensitivity and a 100% specificity [8]. While molecular identification is most reliable, species identification requires sequencing of barcoding genes or the detection of species-specific genetic biomarkers. For instance, both conventional PCR and quantitative PCR-based approaches are developed for species identification of *M. mycetomatis*, *Madurella pseudomycetomatis*, *Madurella tropicana*, and *Madurella fahalii*. These molecular tools, however, are often not available in primary care settings, especially in endemic countries with limited resources [7, 9].

Early diagnosis of mycetoma, especially in remote areas, is essential for enhancing management strategies in endemic regions. Therefore, the implementation of both user- and field-friendly diagnostic tools that do not require expensive instruments and skilled laboratory personnel is conditional [10]. One promising strategy for molecular diagnostics in the point-of-care format, is the use of isothermal DNA amplification technologies such as recombinase polymerase amplification (RPA). This technology has been reported to meet a desirable analytical sensitivity and specificity, exhibit higher tolerance to inhibitory compounds and provide shorter time to results, compared to other nucleic acid amplification tests [11–14]. A significant advantage of RPA is its potential for straightforward implementation in field settings. For instance, the use of RPA allowed the development of a mobile suitcase laboratory for the molecular diagnosis of *Leishmania donovani*. This RPA offered similar sensitivity and specificity as the real-time PCR, but with results six to nine times faster [15].

In 2015, an RPA was developed for *M. mycetomatis* with a reported sensitivity and specificity of 100% based on clinical samples [13]. However, this RPA required purification of the product and visualization using gel electrophoresis, thereby limiting field implementation. A probe-based RPA design will circumvent the necessity of both purification and electrophoresis, greatly improving the turnaround time and field accessibility. This requires an alternative readout method, such as fluorescence, which can be measured by devices such as portable real-time fluorometers. For the fungi *Leptosphaeria maculans* and *L. biglobosa*, such an on-site probe-based RPA was designed with similar sensitivity compared to its real-time PCR counterpart [16].

In this study, we developed a pan-fungal probe-based multiplex RPA for the detection of the fungal ITS region, and to specifically detect the DNA of *M. mycetomatis* and *F. senegalensis*. *M. mycetomatis* and *F. senegalensis* were selected as these are the most common causative agents of eumycetoma, highlighting the relevance of this diagnostic tool in managing mycetoma effectively.

## Method

### Fungal and bacterial isolates and DNA isolation

The collection of 71 fungal and five bacterial isolates of mycetoma causative agents that were used in this study consisted of *M. mycetomatis*, *M. pseudomycetomatis*, *M. fahalii*, *M. tropicana*, *F. senegalensis*, *Falciformispora tompkinsii*, *Trematospheria grisea*, *Medicopsis romeroi*, *Scedosporium boydii*,* Exophiala spinifera*, *Actinomadura pelletieri*,* Actinomadura madurae*, *Streptomyces sudanensis*, *Streptomyces somaliensis* and *Nocardia brasiliensis*. A summary of the number of included isolates is provided in Table 1, and a full overview of all included isolates is provided in Supplementary Table 1. The fungal isolates were maintained on Sabouraud Dextrose Agar (Becton Dickinson, USA) at 37^o^C or 21^o^C (room temperature), depending on the isolate. Bacterial isolates were maintained on BBL™ Trypticase™ Soy Agar with 5% Sheep Blood (Becton Dickinson, USA) at 37 -^o^C. DNA was isolated using a the Fungal/Bacterial DNA MicroPrep kit (Zymo Research, USA). Here, the fungal mycelium was transferred in BashingBead buffer provided in the kit, and lysed using a tissue lyzer (Qiagen, Germany) at 30 Hz for 150 s using five 2-mm metal beads. Next, the DNA isolation was continued according the manufacturer’s instructions. The DNA concentration was determined using the nanodrop for the validation of the probe, and using the Quant-iT™ PicoGreen™ dsDNA Assay Kit (Invitrogen, USA) to determine the limit of detection with increased accuracy, according to manufacturer’s instructions.

**Table 1:** Summary of the included collection

Species	Number of isolates
*Madurella mycetomatis*	44
*Madurella pseudomycetomatis*	4
*Madurella fahalii*	3
*Madurella tropicana*	3
*Falciformispora senegalensis*	6
*Falciformispora tompkinsii*	1
*Trematosphaeria grisea*	5
*Medicopsis romeroi*	3
*Scedosporium boydii*	1
*Exophiala spinifera*	1
*Actinomadura pelletieri*	1
*Actinomadura madurae*	1
*Streptomyces sudanensis*	1
*Streptomyces somaliensis*	1
*Nocardia brasiliensis*	1

### RPA primer and probe design

**Fig. 1:**
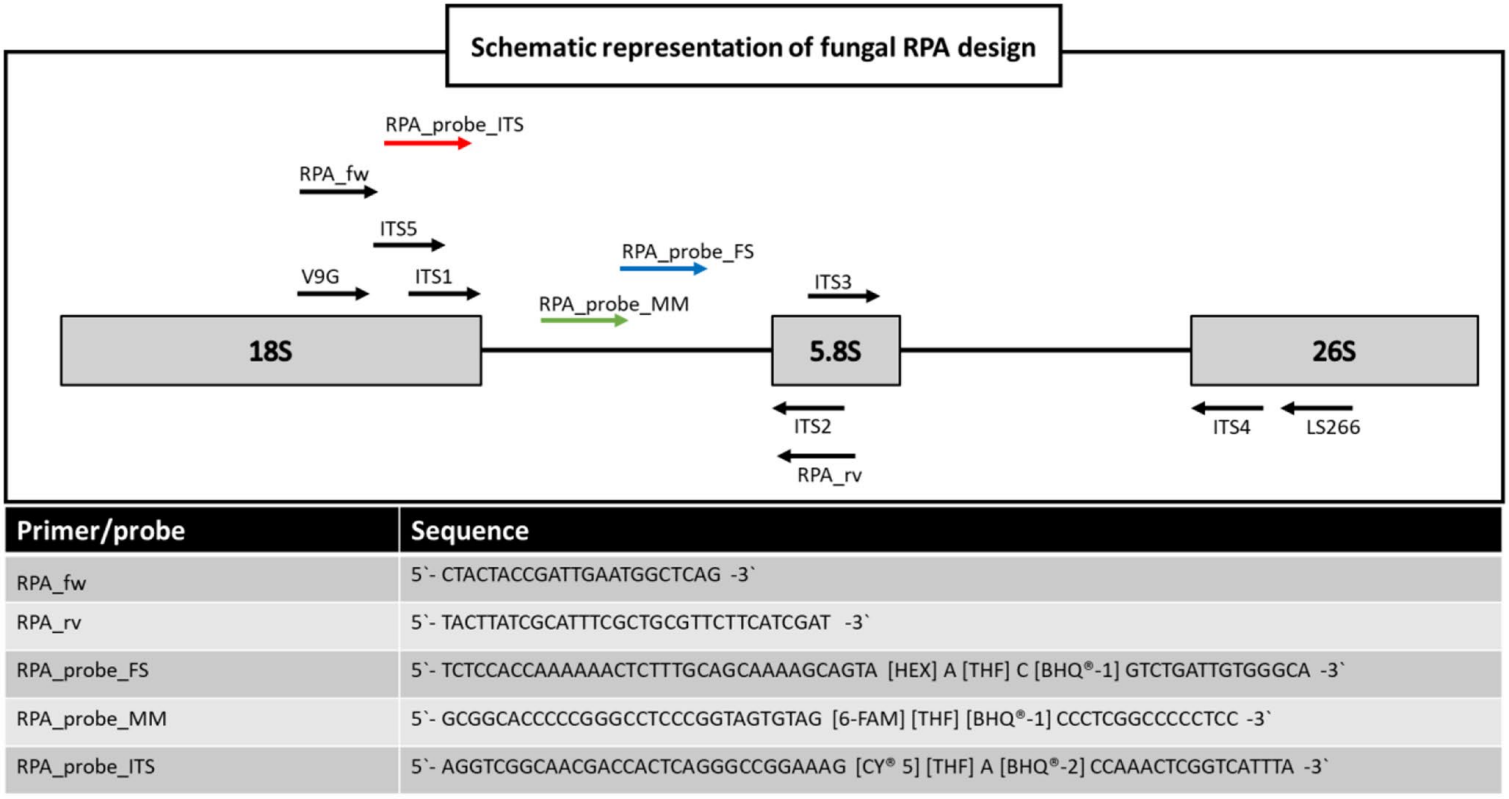
Schematic representation of the fungal RPA design. Listed in the table are the forward (RPA_fw) and reverse primer (RPA_rv) and the three specific probes targeting the conserved region (ITS), the *M. mycetomatis* (MM) specific regions, and *F. senegalensis* (FS) specific region. Commonly used primers for fungal barcoding have been included for reference

The Internally Transcribed Spacer region of eumycetoma causative agents and closely related species were extracted from GenBank (Supplementary Table 2) and aligned using MEGA-X [17]. The sequences were used to identify candidate regions for primer and probe design according to manufacturer’s instructions on RPA design [18]. An overview of the multiplex design and the list of designed primers and probes is shown in Fig. 1. In short, pan-fungal primers and a pan-fungal probe were designed to indiscriminately amplify and detect the ITS1 region of eumycetoma causative agents indiscriminately. Furthermore, probes were designed to detect the presence of the two most common causative agents of eumycetoma specifically, namely *M. mycetomatis* and *F. senegalensis*.

### Primer validation

After the design of the RPA, the primers were validated for specificity against a smaller DNA subset of mycetoma causative agents at an amount of 10 ng using the TwistAmp basic kit (TwistDx Limited, United Kingdom) according to a modified protocol of the manufacturer’s instructions. The TwistAmp basic kit is the standard RPA kit that amplifies DNA, allowing for evaluation of the amplicon. In short, a master mix was prepared consisting of 29.5 µL Primer Free Rehydration buffer, 11.45 µL water, 1 µL forward and 1 µL reverse primers (50 µM). The TwistAmp Basic pellet aliquot was resuspended in the master mix and mixed thoroughly. Following the preparation of the master mix, 2.5 µL magnesium acetate (280mM) was added and mixed by vortexing two for seconds. A 10 µL aliquot of the master mix was transferred to a 96-well plate (Bio-Rad, USA). The RPA was run using a conventional PCR machine (Bio-Rad, USA) at 37 -C for 25 min. The amplicons were cleaned using Zymo DNA Clean and concentrator (Zymo Research, USA) according to the manufacturer’s instructions, and visualized by gel electrophoresis on a 2.5% gel. Amplicons were evaluated to confirm the amplification of the ITS region for all species of interest.

### RPA conditions

The RPA reaction was prepared according to a modified protocol using the TwistAmp exo kit based on the manufacturer’s instructions. The TwistAmp exo kit includes an exonuclease that will cleave double stranded DNA, which allows for the cleaving of DNA-bound probes and release of fluorescent signal. In short, a master mix was prepared consisting of 29.5 µL Primer Free Rehydration buffer, 11 µL water, 1 µL forward and 1 µL reverse primers (50 µM) and 0.6 µL of each respective probe (10 µM). The pellet aliquot from the TwistAmp exo kit (TwistDx Limited, United Kingdom) was resuspended in the master mix and mixed thoroughly. Following the preparation of the master mix, 2.5 µL magnesium acetate (280mM) was added and mixed by vortexing for two seconds. A 10 µL aliquot of the master mix was transferred to a LightCycler^®^ 480 multiwell 96-well plate (Roche, Basel) and mixed with 2 µL of the DNA sample (10 ng) by resuspending three times. The 96-well plate was sealed and placed in the LightCycler^®^ 480 (Roche, Basel), where it was incubated at a constant temperature of 37 ^o^C for 25 cycles, each cycle representing a single minute and a measuring point. The fluorescent output was measured at 465–510, 533–580 and 618–660 nm and analyzed using the Abs Quant/Fit Points in the LightCycler 480 SW 1.5.1 program (Roche, Basel) with an analysis set to 20 cycles.

### RPA validation

The multiplex RPA was validated in three distinct steps. First, the probes were screened against a small subset of DNA samples in singleplex and multiplex to confirm the intended functionality of the probes. Second, the multiplex RPA was screened against a larger DNA collection consisting of different eumycetoma causative agents to evaluate specificity of the probes. DNA from the type strains of actinomycetoma causative agents was included as a negative control. The full list of included samples is listed in Table 1. Third, the limit of detection (LOD) was determined based on a 10-fold dilution series ranging from 10 ng to 0.00001 ng (10 fg) of DNA isolated from *M. mycetomatis* strain MM55 and *F. senegalensis* strain CBS 132272. Here, the Quant-iT™ PicoGreen™ dsDNA Assay Kit (Invitrogen, USA) was used according to the manufacturer’s instructions to determine the DNA concentration of the DNA stocks accurately. For all three probes, the LOD was determined by performing three technical replicates in both singleplex and multiplex. The data was analyzed as described above and the Cp values of each sample were plotted against the respective DNA concentration and visualized for each of the respective probes.

### Costs of the RPA

Based on our methodology, the number of reactions that can be performed was used to calculate the costs. The costs of the primers and probes are determined based on the time of purchase. The listed price of the items, was the price indicated on the day of the online search. The RPA kit was obtained from TwistDx for € 448.91 [19]. The primers and probes were obtained from Eurogentec Custom Oligo synthesis, prices listed in Table 2. The calculation was based on the method described in the validation process, using the Roche Lightcycler 480. In this machine amplification was performed on plates purchased for € 433.99 per 50 plates [20]. Additionally, the theoretical cost of using single tubes was incorporated as alternative to using a 96-wells plate. Labor costs, and the costs of other consumables, were not included in the cost-calculation.

## Results

### Primers successfully amplified the DNA of different fungal species

**Fig. 2:**
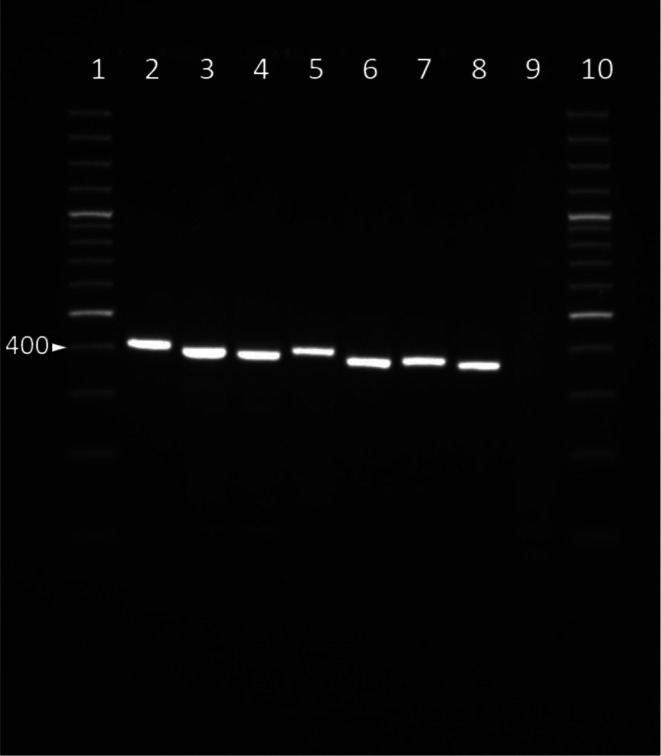
Primer validation of RPA forward and reverse primers. *M. mycetomatis* CBS 109801T (lane 2), *M. fahalii* CBS 129177T (lane 3), *M. pseudomycetomatis* CBS 129176T (lane 4), *M. tropicana* CBS 201.38T (lane 5), *F. senegalensis* CBS 132257 (lane 6), *F. tompkinsii* CBS 200.79 (lane 7), *M. romeroi* CBS 252.60T (lane 8), negative control (lane 9), and 100 bp plus ladder (lane 1 and 10)

To validate the designed RPA primers, a subset of different eumycetoma causative agents was evaluated. Gel electrophoresis was used to visualize the resulting products. With the RPA primers, an amplicon was obtained for all eumycetoma causative agents included in the panel (Fig. 2). For *M. mycetomatis* the size of this amplicon was 407 base pairs, and for *F. senegalensis* 376 base pairs. Due to natural differences in the targeted ITS1 region between the species, the amplicon size was different for the specific causative agents, as seen in Fig. 2. Some alternative products were found for all species.

### RPA probes discriminate between causative agents

**Fig. 3:**
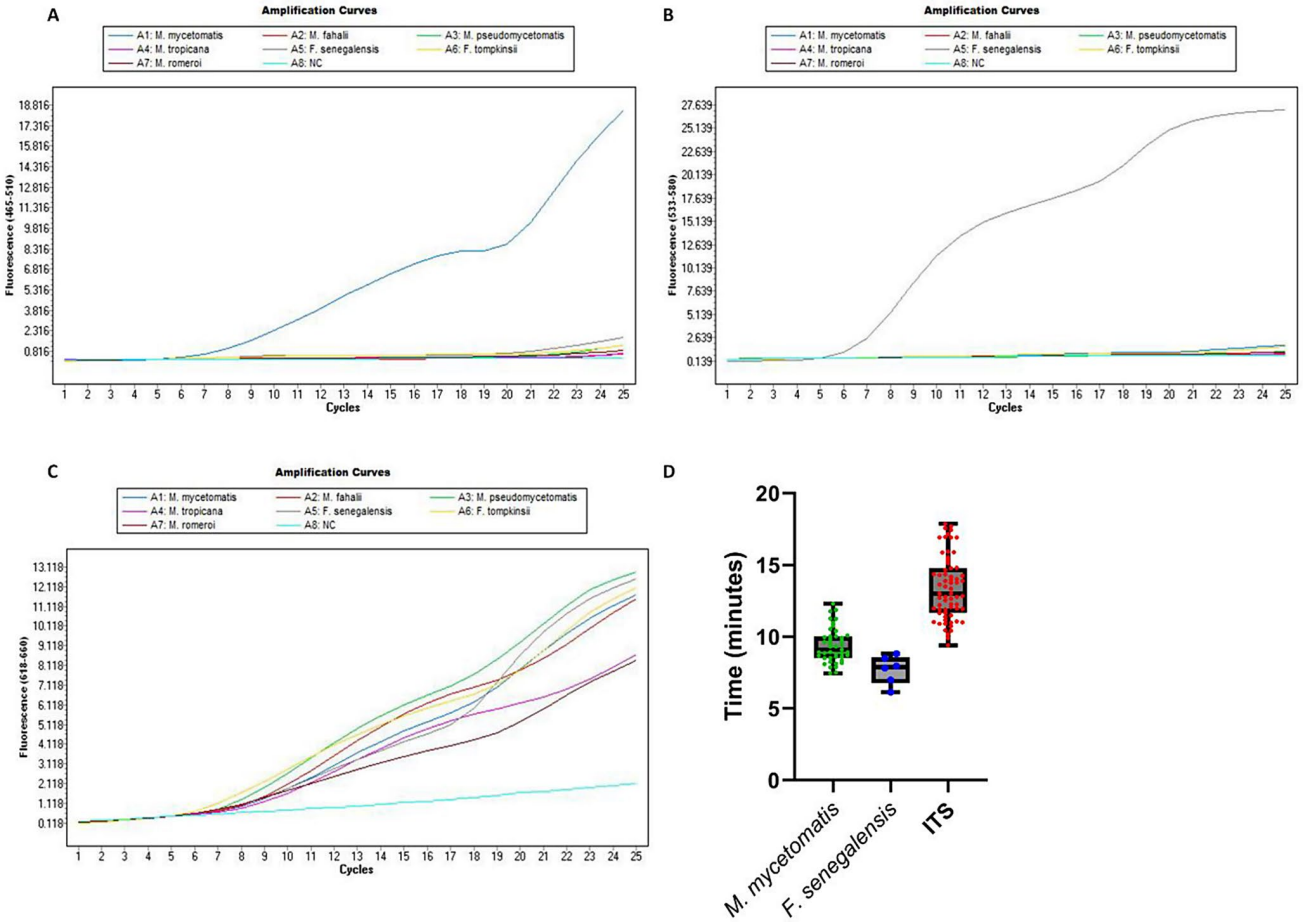
Validation of the probe specificity determined against mycetoma causative agents. **A-C** RPA Probe specificity against 10 ng of DNA derived from a small subset of eumycetoma causative agents. The small subset consisted of *M. mycetomatis* (CBS 109801T), *M. fahalii* (CBS 129176T), *M. pseudomycetomatis* (CBS 129177T), *M. tropicana* (CBS 201.38T), *F. senegalensis* (CBS 132257), *F. tompkinsii* (CBS 200.79), and *M. romeroi* (CBS 252.60T). Fluorescent signal plotted against cycles, for which each cycle represents one minute. NC is the elution buffer supplied with the Zymo DNA isolation kit, used as a blank sample. **(A)** Fluorescent signal measured at 465–510 nm corresponding to the FAM fluorophore of the *M. mycetomatis* probe. **(B)** Fluorescent signal measured at 533–580 nm corresponding to the HEX fluorophore of the *F. senegalensis* probe. **(C)** Fluorescent signal measured at 618–660 nm corresponding to the Cy5 fluorophore of the ITS probe. **(D)** RPA Probe specificity against 10 ng of DNA derived from 71 different eumycetoma causative agents was evaluated in the RPA. The time to positive signal was plotted for the different probes. The *M. mycetomatis* probe resulted in a positive signal for all 44 *M. mycetomatis* DNA samples, ranging between 7.46 and 12.31 min and a mean of 9.3 min. The *F. senegalensis* probe resulted in a positive signal for all six included *F. senegalensis* DNA samples, ranging between 6.14 and 8.83 min to a positive signal with a mean of 7.6 min. The ITS probe was positive for all 71 included fungal DNA samples ranging between 7.88 and 17.87 min and a mean of 13.1 min

RPA probes were first validated against the same subset of eumycetoma causative agents (Fig. 2). As shown in Fig. 3A, a positive signal with the *M. mycetomatis* specific probe was found for *M. mycetomatis* (CBS 109801T), and not for any of the other *Madurella* species. The *F. senegalensis* specific probe only gave a positive signal with *F. senegalensis* (CBS 132257), notably no signal was detected for the closely related species *F. tompkinsii* (Fig. 3B). The ITS probe resulted in a signal for all included isolates (Fig. 3C). The RPA was validated against a larger subset of DNA derived from different mycetoma causative agents. The complete overview of these results is summarized in Supplementary Tables 3, and a condensed overview is shown in Fig. 3D. The *M. mycetomatis-*specific probe resulted in a positive signal for all 44 included *M. mycetomatis* DNA samples within 7.46 to 12.31 min, but not for any of the other tested eumycetoma causative agents. Similarly, The *F. senegalensis-*specific probe only resulted in a positive signal for the five included DNA samples derived from *F. senegalensis* after 6.09 to 8.83 min, but not for any of the other DNA samples. For the ITS probe, all fungal-derived DNA samples resulted in positive signals after 7.88 to 17.87 min. No positive signals were detected for DNA derived from the actinomycetoma causative agents: *A. pelletieri*,* A. madurae*, *S. sudanensis*, *S. somaliensis* and *N. brasiliensis*.

### RPA detection limit

**Fig. 4:**
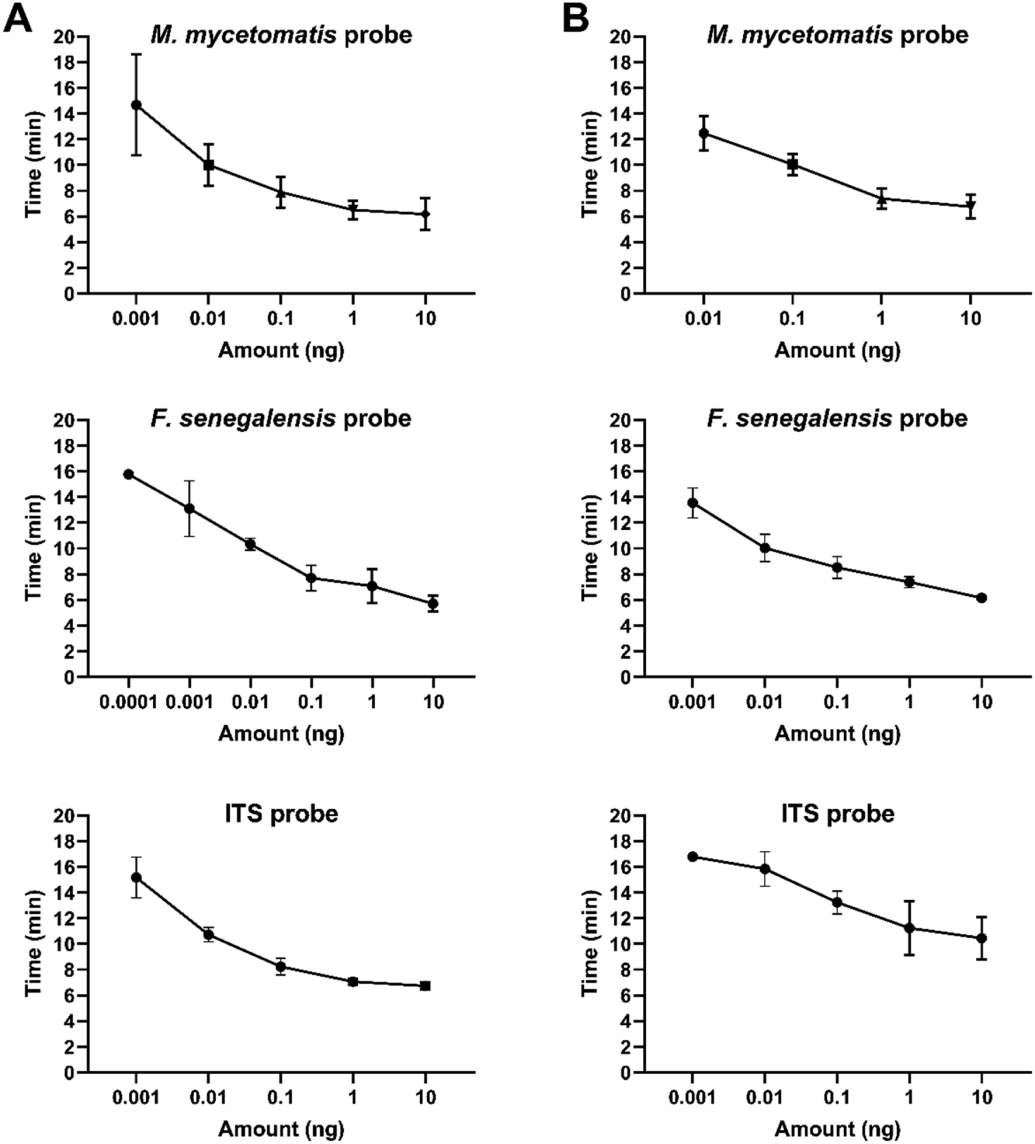
Limit of detection of the probes plotted against the time in minutes until a positive signal is detected. **A** Probes tested in singleplex against DNA derived from *M. mycetomatis* stain MM55 and *F. senegalensis* strain CBS 132272, ranging from 0.0001 ng and 0.001 ng, to 10 ng, is plotted against the time until a positive signal is determined. **B** Probes tested in multiplex against DNA derived from *M. mycetomatis* stain MM55 and *F. senegalensis* strain CBS 132272, ranging from 0.001 ng and 0.01 to 10 ng, is plotted against the time until a positive signal is determined

The limit of detection of all three probes, both singleplex and multiplex, was determined by amplifying the different concentrations DNA of *M. mycetomatis* MM55 and *F. senegalensis* CBS 132272, ranging from 10 fg to 10 ng. In singleplex, 0.001 ng, or 1 pg, of fungal DNA was reliably detected using the *F. senegalensis* probe and 0.01 ng, or 10 pg, of fungal DNA was reliably detected using the *M. mycetomatis* and ITS probes (Fig. 4 and Supplementary Table 4). In multiplex, the amount of fungal DNA that was reliably detected decreased by one dilution step. For the *F. senegalensis* probe 0.01 ng, or 10 pg, of fungal DNA was reliably detected, and for the *M. mycetomatis* and ITS probes 0.1 ng, or 100 pg, of fungal DNA were reliably detected (Fig. 4 and Supplementary Table 5). Based on an average *M. mycetomatis* genome size of 36.7 Mbp, this equals 252.44 copies of the genome [21].

### Costs of the multiplex

The reagents used in the execution of this multiplex RPA are shown in Table 2. Based on these reagent costs and on one pellet al.iquot used to prepare four multiplex RPA reactions, the calculated reagent costs of a single sample are €1.35. Using the Roche Lightcycler 480, the cost for a single reaction increases tremendously, due to the required 96 well plates costing €8.68 if a single sample was tested on one plate. Therefore, the total costs of the RPA multiplex in the described set-up adds up to €10.03, excluding additional costs such as pipet tips, equipment maintenance, the initial purchase of the machine and labor. As performing the RPA in a 96-wells plate is not sustainable for field use, the hypothetical cost of testing a single sample has also been calculated in case the EasyStrip™ Plus Tube Strip with Attached Ultra Clear Caps would be used. Here 250 strips of 8 tubes could be obtained for €426, potentially lower the overall costs to €1.56 to test a single sample [22].

**Table 2:** Costs of the multiplex RPA. The costs are shown for testing a single sample. When Preparing the reagent mix, the entire reagent tube needs to be prepared, but four reactions can be performed from a single tube. In the described methodology, testing a single sample requires ¼ of a TwistDX reaction tube. Both the minimal costs and the optimal cost of the test, are reflected in the costs analysis of this test

Product	Price/product	Amount	Work concentration	Units/product	Units/reaction	Nr. Of reactions possible	Price/reaction	Price/1/4 reaction
RPA exo kit	€ 448.91	1 kit		96 reactions	1	96	€ 4.68	€ 1.17
Probe Mm	€ 441.81	40 nmol	10 µM	1040	0.6 µL	1733.33	€ 0.25	€ 0.06
Probe Fs	€ 481.43	40 nmol	10 µM	1486	0.6 µL	2476.67	€ 0.19	€ 0.05
Probe ITS	€ 516.65	40 nmol	10 µM	1174	0.6 µL	1956.67	€ 0.26	€ 0.07
Primer Fw	€ 5.98	40 nmol	50 µM	6500	1 µL	6500	€ 0.00	€ 0.00
Primer Rv	€ 8.58	40 nmol	50 µM	6080	1 µL	6080	€ 0.00	€ 0.00
**Total reaction costs**							**€ 5.39**	**€ 1.35**
lightcycler plates roche	€ 433.99	50 plates		50	1	50	€ 8.68	€ 8.68
EasyStrip™ Plus Tube Strip with Attached Ultra Clear Caps	€ 426.00	250 strips of 8 tubes		2000	1	2000	€ 0.21	€ 0.21

## Discussion

Diagnosis of eumycetoma remains challenging particularly in remote areas, and implementation of field-friendly diagnostic tools is crucial to enhance the management of mycetoma in these regions [10]. To address this need, we developed a multiplex probe-based RPA assay capable of detecting DNA from fungal causative agents of mycetoma, and to discriminate between the most common eumycetoma causative agents *M. mycetomatis* and *F. senegalensis.* The isothermal amplification and fluorescence-based readout of this probe-based RPA facilitates real-time monitoring of DNA amplification at a constant temperature. The RPA was validated against 44 *M. mycetomatis* isolates and 6 *F. senegalensis* isolates. Unfortunately it was not possible to include a higher number of *F. senegalensis* isolates for the validation, as these are currently not available in our own collection or in commercial culture collections.

Currently, accurate identification of the eumycetoma causative agents of eumycetoma typically relies on molecular techniques, such as sequencing or discriminative PCRs [8, 23]. Such PCRs are designed for the diagnosis of *M. mycetomatis*, *M. pseudomycetomatis* and *S. boydii*, and a real time PCR is available for discrimination between the *Madurella* species [24–27]. However, these techniques are generally not suited for endemic regions, as the equipment needed is bulky and, in the case of conventional PCR, gel electrophoresis is needed for visualization of the results. Recently, field friendly real-time PCR machines have been introduced such as the Biomeme Franklin, a handheld, battery-operated, portable thermocycler. Using this system, a freeze dried reagent-based PCR assay developed for the molecular diagnosis of Buruli ulcer, reported a 97–100% sensitivity and 94–100% specificity in clinical samples across three reference laboratories in endemic countries [28]. Although the introduction of such real time PCR machines may mitigate some of the drawbacks that limit the integration of real-time PCR in resource-limited settings, the RPA still offers advantages. These advantages include the single temperature operation, tolerance to common PCR inhibitory factors, and, most notably, the shorter turnaround time [29]. The latter is especially important as field-friendly tools generally have limited capacity, and shorter assay duration enables higher sample throughput. For instance, the detection of *Madurella* species with real-time PCR has a run-time of ~ 60 min, while the run-time of the multiplex RPA is 20 min [24]. We report mean times to positive result of 9.3, 7.6 and 13.1 min for the *M. mycetomatis* probe, *F. senegalensis* probe and ITS probes respectively. These turnaround times are in line with previous studies, which report the detection of DNA within 15–30 min for other pathogens such as *L. maculans*, *L. biglobosa*, *L. donovani*, *Escherichia coli*, and HIV [12, 15, 16, 30]. Interestingly, we find some internal differences between our designed probes, and the ITS probe, which requires 4–6 min longer before a positive signal is detected. This is particularly interesting since the ITS probe targets a different region within the same target as the *M. mycetomatis* and *F. senegalensis* probes, and therefore, no difference in the target of the probes is expected. Furthermore, this finding seems to be irrespective of probe competition, as this finding is not limited to *M. mycetomatis* or *F. senegalensis* derived DNA samples. As to the extent of our knowledge this finding is not reported for the RPA, we postulate that this is likely due to differences in the binding affinity of the probe and the accompanied lower reaction efficiency.

The difference in time to positive result, however, does not seem to influence the limit of detection (LOD). Here, we report a detection limit for the multiplex RPA of 0.01 ng of DNA, with a specificity of 100%. These findings are in a similar range as previously reported for *M. mycetomatis* by Ahmed et al., with a LOD of 0.2 ng for the RPA, and 0.5 ng for the LAMP [13]. Recently, Yoshioka et al. reported a LAMP design that detected the DNA of *Madurella* spp. up to 1 pg within 60 min, this test was also able to distinguish between *M. mycetomatis* and *M. fahalii* [31]. While a low limit of detection is desirable, the lowest amount of fungal DNA isolated from one grain has been reported to be 30 ng, which is comfortably within the limit of detection of the RPA [32]. However, in our study, standardized DNA derived from cultures was used, which is not necessarily reflective of clinical samples. Grains as starting material for the molecular detection of eumycetoma causative agents has been successfully used before [23, 32, 33]. PCR, in combination with ultrasound-guided fine needle aspiration cytology, obtained 97% sensitivity and 100% specificity on grain samples, indicating that a molecular-based detection method of grain material is a reliable way to identify the causative agent [34]. Although the previous *M. mycetomatis-*specific RPA was reported to equal the performance of the PCR in clinical samples, further evaluation of clinical material is required for the RPA reported in this study [13].

The cost of reagents for the RPA calculated conform the presented methodology was €1.35 for one single sample. Naturally, the read-out system should be taken into consideration as well. In our set-up, the use of the Roche Lightcycler required specific 96-well plates, driving the theoretical price to test a single sample up to a total of €10.03. This, however, depends on the number of reactions, as the more samples tested in a single run, the lower the overall costs will be. Furthermore, the use of a different read-out system that requires single tubes (e.g. EasyStrip™ Plus Tube Strip with Attached Ultra Clear Caps, Cat#:AB2005, Thermo Scientific™), would also potentially lower the overall costs to €1.56 to test a single sample [22]. While for mycetoma the costs of currently available molecular tests have not been analyzed, the costs of the RPA multiplex is in line with a cost analysis reported for genetic testing using PCR [35]. In comparison, an extensive cost-analysis concluded the cost per unit of providing COVID-19 rRT-PCR tests during early stages of the pandemic was estimated at $7.5. While this analysis included a wide variety of fixed and variable costs, it was noted that purchasing laboratory consumables accounted for up to 87% of the costs [36]. Notably, our analysis did not include labor-costs, which are also influenced by the number of samples tested in a single run and the demographic location where the test is performed [36]. Altogether, the costs of this multiplex RPA are within the limits of affordability for nucleic acid testing, as suggested in the REASSURED criteria [37].

In conclusion, we report the development of a multiplex RPA that can accurately detect up to 0.01 ng of fungal DNA within 20 min, and can discriminate between the two most common causative agents of eumycetoma, *M. mycetomatis* and *F. senegalensis*. Furthermore, to the extent of our knowledge, this is the first reported design of direct molecular detection of *F. senegalensis.* While clinical validation is required, this tool holds promise for enhancing diagnostic capabilities in eumycetoma endemic regions, paving the way for improved patient management and treatment outcomes.

## Electronic supplementary material

Below is the link to the electronic supplementary material.

Supplementary Material 1

Supplementary Material 2

## Data Availability

No datasets were generated or analysed during the current study.
